# Proceedings of American Dental Association

**Published:** 1875-02

**Authors:** 


### SELECTED ARTICLES.

---

### ARTICLE III.

### *American Dental Association.*—CONTINUED.

FOURTH DAY.—*Morning Session.*

The Association was called to order by the President.

The report of the Treasurer was read and accepted.

The following resolution, introduced by Dr. Shepard, was passed unanimously:

*Resolved,* That the American Dental Association fully appreciates the promptness with which Dr. S. S. White responded to the requests of the dental profession, to take the management of the suit for the ascertainment of the rights of the public in regard to the use of vulcanite for dental purposes, and would respectfully request him to continue his valuable aid in carrying up the case for final adjudication by the Supreme Court of the United States, pledging him our moral and material support in defraying the expenses that may be incurred in the future.

A volunteer paper by I. Douglass, of Michigan, was read by Dr. Cushing, giving an account of two cases of apparent death from chloroform. The first case was that of a patient having palpitation of the heart, but no organic disease. After having twenty teeth extracted, the patient recovered consciousness, but immediately upon ascertaining the fact that the teeth were out, she relaxed and ceased to breathe. A battery which was near was immediately called into requisition, and with the first application the respiration and pulse appeared, and the patient made a speedy recovery. Fear had undoubtedly acted as an antidote to the chloroform, and the removal of that caused the syncope.

The subject of "Dental Education" was then taken up, and the report of the committee, written by Prof. McLain, of New Orleans, was read by Dr. Knapp. We give a synopsis of the report, as follows:

Of late years a gradual loss of confidence, amounting almost to distrust as to the efficacy of dental colleges, has taken place in the minds of leading practitioners; it is believed that graduation has become too facile, more from rivalry among the colleges than incompetency of the professors. No body of men has made greater pecuniary and personal sacrifices than these professors, laboring and expending money without either present or hope of future emolument. But the colleges have failed to meet just ex pectations; the profession looks to the colleges for the training necessary to place it in accord with the spirit of progression of the age. The colleges have been somewhat remiss in elevating the standard proportionally to our advance. One cause is the custom of examinations being conducted by the professors, who may allow sympathy to relax their rigidity, if not lead to partiality. Another cause is the practice of graduating students on attending two sessions, which is equivalent to making dentists in eight months of actual study. So limited a period is insufficient, coming unprepared as the student often does. Mechanics would consider a two-years' apprenticeship insufficient to learn a trade; how can we expect that a knowledge can be gained in that time of a profession, involving high skill and acquaintance with several abstruse branches of science? The wonder is that the graduates are ever more than mediocre. Lack of previous education prevents the colleges from adopting a higher curriculum. Statutory enactments will not cure the evil as to colleges; but regulating laws would shut out the grosser and more reprehensible elements. What is needed is that the profession furnish the colleges with better material, and they should not accept pupils who have not a good preliminary education and natural fitness, and should obligate them to study three years and pass through a dental college. The colleges should confide the examinations to boards outside of the institutions, and thus avoid the charge of favoriteism. They should lengthen their courses to five or six months, and require an attendance of from three to five sessions as a condition of graduation. These measures are a matter of self preservation with the colleges, for at the present rate of increase, the profession will soon be overrun, and its remunerative prospects destroyed, and the reproach incurred would affect them injuriously. The first college adopting these measures might temporarily suffer, yet in the end it would be compensated and would gain the support of the profession.

Dr. Keely followed with an additional report, of which the following is a synopsis:

Dental surgery is a legitimate speciality of medicine, and should assume its true functions in the healing art, by the principles of which its processes should be controlled. Its basal elements are a thorough study of the whole organism,—and such knowledge is now required. Mechanical skill with a few general ideas are not now a preparation for dental practice; knowledge is power in this as in other branches. The medical profession has been cold toward us, and the fault is with it. Let the dentist demonstrate his worthiness, and this will disappear. Preliminary literary culture is demanded. The mind and heart must be trained.

Dental practice needs manhood as its basis. The college diploma is often a fraud, but so transparent as to deceive only the ignorant. What is truly represented by it is the least that should satisfy the student. It is of little importance whether the medical knowledge is taught in a medical or a dental college if it is well taught, and the preparation honestly and thoroughly made. The student is then prepared to appreciate his special branch of study, and his time will be saved for its higher elements. Impatient aspirants may turn away in disgust, and seek for a shorter road. Let such remember that the time for success in this way has passed; any scientific calling demands far more elaborate preparation than in the last generation. Daily requirements of practice call for a more advanced and scientific knowledge. A young man who rashly assumes these duties, condemns himself to a meagre share of the benefits of his calling, and exposes his patients to the consequences of his ignorance. The student should be under the direction of a preceptor who is competent, and of recognized skill, who will require from three to five years' study. A sensible preceptor will unfold to the aspirant his deficiency in any element of success, and give broad views of professional duty; will moderate his impatience to assume those duties. He will point out the vast number of ill-educated men sent forth from the rival medical schools, and deprecate this policy in the dental colleges. To swell the number of graduates, the standard of attainment must be lowered. There are schools which do not yield to this temptation, and their diplomas stand high, because they indicate a definite amount of preparation. The error of establishing a dental college in every great city should be avoided, and effort should be concentrated at a few points, say three or four, which will be ample for our whole country,—one east, one west, one south, and, when the time arrives, one on the Pacific coast; for this number an endowment could be easily secured. The professors are at present poorly paid, and obliged to eke out their subsistence by professional labor. This interferes with the value of their instructions, which are sufficient to tax fully a first-class mind. No professor holds his position except at a pecuniary sacrifice, which should not be required. Our profession owes to them a debt of gratitude. This policy of concentration will ignore local interests, and provoke recrimination; but truth is ascertained only after sharp altercation. We should hold to the policy when its necessity is recognized in spite of the clamor raised by interested parties,—its necessity is obvious. By limiting the number of colleges, an endowment can easily be secured, and each professor can receive a competent salary; but by dividing our means on a number of starveling projects, we shall inflict the same evils upon our profession which our literary and medical institutions are now deploring.

The subject was then opened for discussion.

Dr. John Allen said that dental education embraced more than that education which comes from others; it also embraced that which each man gives himself. The failure of dental colleges to discharge their duties, or rather of graduates to meet the requirements of practice, is due to the want of this second education. They settle down into mediocrity because they have a diploma. It is self-education that enables men to get beyond others. A celebrated painter said he mixed brains with his colors; we should mix brains with our work. The artificial branch of dentistry has been neglected, and it is lower to-day than it was thirty years ago, though on the whole our profession stands higher.

Dr. Walker said no perfect brain is found in an imperfect body. Dental colleges should go a little farther than they have been going. We should have a knowledge of the system beyond the mouth, which will enable us to avoid breaking down and spending months to recuperate.

Dr. Taft. There are many obstacles in the way of thorough dental education. One rests with preceptors. Great care should be used in selecting students; inheren ability and preliminary education should be considered. They should have a good general education and a knowledge of the fundamental principles of medicine and surgery. A student going first to the dental college has no appreciation of what he is about to do. Let him understand what is before him. Do not say to him that a year or two of private pupilage and two courses in a college will fit him to practice dentistry. Some societies have resolved that their members should not receive students for a less period than three years and two courses in a dental college. Our best students come from the offices of men who give them a thorough training. This body should go in advance of the local societies, and should make a clear pronouncement upon this subject, and no college in the country could ignore it.

Dr. Thomas. Some admit students to their offices, for pecuniary considerations, that have no business there. It is cruel to advise a young man to come into the profession in less than seven years. Students should go directly to a dental college, and not into an office; because no two practitioners have the same ideas. If there are men competent to take students, they are not the men who will do it, and for this reason he wonld prevent dentists from taking students.

Dr. Stockton. We live in a busy age; it takes but a little while to become a *grandfather in dentistry;* that is, to send out a student, who in a short time will have sent out his student. The Ohio Dental College once required as a qualification for students that they should know something of the English language; but the pressure was so strong that this has been swept from their books. New Jersey has a law requiring an examination before a board; and the board rejects every young man who is not thoroughly qualified. If none could enter the profession except a graduate, we should soon place our profession where we wish to have it, and where we must insist that it shall come.

Dr. Bogue. In New York there is a law and a board of censors. Does not conceive that the multiplication of State laws is going to affect graduates. The public opinion of dentists moulds the action of colleges. We have no standard. One school may take a stand that all will approve; another will sell its diploma for twenty-five dollars. We should take such a course that they will take a different stand from what they now do.

Dr. Butler. Diplomas should mean something. They are too cheap,—there are too many graduates. Would like students to graduate both at a dental and medical school and then they would not have too much knowledge. Does not think it is policy now to send a student to a dental school, no matter how good. Fault is found with both schools and graduates, yet there is a disposition to multiply colleges. They ought to be simmered down to two or three, and faculties put in that are good for something, and endowed with a sufficient fund to give them good teachers. The best schools are those that give the students ample clinical instructions. The teaching of mechanical dentistry amounts to little,—we are not so far advanced in that department as we were thirty years ago.

Dr. Allport. A great deal can be said about this question, but the thing is to say something practical. It is easy to blame the schools, but not so easy to suggest some practical plan whereby a thorough education will be more general. There is a desire that dentistry should be regarded as a specialty of medicine, but the present course of instruction in either private offices or colleges is not the best to secure that end. The same course of study should be pursued that other specialists in medicine go through with; books are studied and lectures attended for three years; and the extra time is devoted to the study of the special studies; and these cannot be pursued until a general knowledge of medicine is obtained. The student of dentistry, on the contrary, simply takes a partial course of reading, and commences work in the laboratory; thus studying a profession and learning a trade all at once. No one expects a trade to be learned in less than three years' apprenticeship.

Certainly even mechanical dentistry requires as mueh preparation as that; and to properly practice all branches, a man must learn both a trade and profession in that time. The study and practice should be divided. Let the mechanical dentist confine himself to that branch, and let the dentist who treats diseases of the teeth become fitted as a medical specialist in a medical college; let him breathe the atmosphere of medical teaching; then we shall have done the same that is done to make ophthalmotologists and aurists. Designate mechanical dentists as you please, but drop the word *dentist* as soon as you can. We may have "dentologist" to designate those who operate on the natural teeth; the others may be called what you please, as "dentificer" or "dentician," but let them be called by different names, and soon they will be so recognized by the public, who will be better served than by the present practice.

Dr. Morgan is the oldest graduate belonging to this body, and feels entitled to be heard. There are entering the profession one thousand two hundred men each year, and of that number perhaps two- hundred and fifty attend lectures. If the students can be got into the colleges, it will be a great advance. Admits that the colleges are not what they should be. Much stress has been laid upon the time required. Thinks that when a student can pass the examination he should have his diploma, whether in one year or ten. In some institutions that is the mode of procedure. In the University of Virginia there are only three professors, yet a diploma from that institution is the highest recommendation a man can have in the South. They examine at the end of one, two, and three years, and when a man earns his diploma he gets it, on the ground of qualification, and not length of time. If we can get the dentists of the country connected with the associations, we shall remedy some defects in education. In Tennessee there are two hundred and fifty dentists, and only fifty belong to associations; the others are beyond our reach, do not take our literature, never mix with the profession, but squat down in the little towns. If we could bring them in contact with the more advanced in the profession, there would be less difficulty about students. We were among the first to require every student to give at least two years' time, and graduate before they proposed to engage in practice. It has had a wholesome effect, though it has not been lived up to.

Dr. Wetherbee. Public opinion is arrayed against the advance of the profession. The larger part of the people are of moderate means, and call on the first man whose shingle they meet. There are many practitioners who are not properly instructed in the mechanical department; you cannot divorce this department from the operative generally. Some practitioners keep their students for years and never instruct them an hour, or allow them to see a tooth filled. No respectable dentist should take a student for less than three years, and should require him to agree to attend a college at the end of that time and graduate. This association is a stumbling block to our advance. It is composed of graduates and non-graduates. Change the constitution, and allow no delegate to come here who is not a graduate, and you will have set an example to the whole country.

Dr. McQuillen. In listening to the annual criticisms of dental colleges, in our national association, I am forcibly reminded of an utterance of that genial wit and humorist, the "Professor at the breakfast-table," wherein he says,—"When nature invented, manufactured, and patented her authors, she made critics out of the chips that were left." It takes time, labor and thought to write a good book, or to found, manage, and maintain a college; but with a breath or stroke of the pen slight faults ean be exaggerated into great defects, and unfounded assertions of unfaithfulness to duty made without due examination of their anthenticity. No one can be more conscious of the shortcomings and deficiencies of dental colleges than those who are managing them, and endeavoring, so far as they can with limited means and inadequate support, to increase their sphere of usefulness. Do gentlemen appreciate the trouble, time, and expense that those who are engaged in teaching are subject to? Would it not be well, in addition to criticising these efforts, they would aid in the cause of education by contributing money towards endowing the colleges; or, better still, spend their time and money in establishing and maintaining dental colleges, and thus show the profession how much better they could do the work? Why not criticise private preceptors in their unquestionable failure to perform their duty? For the vast majority of accessions to the ranks of the profession come from private offices. The curriculum of instruction in the dental colleges, in place of being lessened, has been enlarged. Formerly a winter course of four months constituted the annual term; now, in addition to that, in some of the colleges, there are spring and fall courses of lectures, while the dispensary and laboratory are open all the year, where every opportunity is afforded for acquiring a practical knowledge of the profession without additional charge to the student. The standard of graduation has also kept pace with this. Speaking not from isolated cases or a limited experience, but as one having had every opportunity of observing the men who have attended lectures in the medical and dental colleges in Philadelphia during the past twenty-five years,—the class of students entering and graduating from the dental colleges now, instead of being inferior to those of former years, are vastly superior in mental culture, for many of them have enjoyed every advantage in scholastic opportunities in the best universities of our own and foreign lands, prior to matriculating in our institutions. We are told that there should be fewer schools; that four would suffice, one in the east, one in the west, one in the south, and one on the Pacific coast. Has the gentleman considered the question in all its bearings? Colleges are established not merely to supply the wants of the present, but the demands of the future; not only to educate those who are to serve, in our land, forty millions of people in the present, but five hundred millions in the future. Again, if all engaged at present in the practice of dentistry who need educating, and the yearly additions to the ranks of the profession who come in from private offices without an education, were forced by State laws or an enlightened public opinion to obtain a collegiate education, the dental colleges now in existence could not accommodate them; indeed, with the present facilities, the best arranged institutions in the country could not give that practical instruction which is such an important element in dental education to more than one hundred students; whereas, on account of the different methods of instruction in medical colleges, five hundred to one thousand men can be taught the theory and practice of medicine and surgery.

A most melancholy spectacle is presented by some of our fellows who are constantly whining about the non-recognition of the profession. The question naturally suggests itself,—is the fault with the profession or the individual? A man of culture, ability, and executive capacity has no occasion to complain of indifference to his rights on the part of others, and it is the influence of such men that gives character and tone to the profession in which they are engaged.

Dr. Crouse. To improve the profession we must improve its material; but he does not agree as to the superiority of graduates over others, Their average is very little above those outside. There is something wrong either with the material or the education when men associate themselves with the worst mountebanks in the country. There are some earnest laborers in the colleges; but the fact that when one college springs up in a city, another must spring up, shows that it is a kind of quackery. One set of men say that another set shall not be above them in being called college professors. Colleges can't educate one hundred properly. He would not send a student to any college, but would give him his dental education, and would send him to a medical college. A dentist should be a great deal more than educated as a manipulator, and that can be done a great deal better in an office than in a college. We cannot expect to be recognized by scientific men, until we become scientific. To make dentists you must have the right kind of preceptors and the right kind of material.

Dr. Atkinson. Dental education is really very far off, if we have to go back through surgical, medical, and classical, to academic education before we can get it. Medical education is the basis of dental education. He has his ideal, but we have to take things as they are. Who of us that has come through all the painful labor, don't know the difficulty of getting what little education we have? If we were required to produce a voter twenty-one years old instanter, with a nice beard on, he don't know how it could be done; we will have to go back to the good old way, begin right, and very likely it will come out right. The mechanical aspect of dentistry, like surgery, enables us to perceive when we have coincided with law or opposed it. Medicine makes mistakes, and the grave covers them. The self-sufficient iconoclast tears valuable organs from their location, and puts them out of the way. I am with and against every man who has spoken; with him so far as he comes up to my conception of the truth; and when he don't I yearn to illuminate him, because I know I have the truth. Ain't *we* the gentlemen, and *they* the boors; ain't *we* the Christians and *they* the vandals the world over? What is education? It is mental feeding and nothing else; it is knowing what kind of food to eat. Sometimes I wish that there were no such things as diplomas. If there must be, do have them say what they mean, and mean what they say. Many in this room have them who never attended a single lecture, and yet they are worthy of them. They do not go through the right way, they say. The brave men who have practiced the self-education spoken of by Dr. Allen,—did they make themselves worthy? No; the blessed love of the fountain of light made them what they are; all they know was received at a time when they were in a receptive condition, and were hungry to know, and the divine grace came in and illuminated their understanding, and revealed to them the truth. When we get hungry for a breath we take it; so when we want education very bad we get it,—where there is a will there is a way.

I wish there were nothing but certificates of advancement. Graduation will leave us but babes; we are not complete in our best apprehensions. Diplomas as a rule are a damage, for they entrap a man into the idea that he has been recognized, and has finished. That is not the intention. It is simply an admission fee to the show,—to where the divine grace may come in and possess him,—make him luminous, and perceive the truth, and then he will embrace it; for there is not a sinner on the earth that will go the way of darkness by preference, the whole pulpit to the contrary notwithstanding, and I am a *pulpiter* too. (Applause.) I am not ashamed of the meanest outside dentist as compared with the meanest outside medical man. I will cast my lot with the dentists; they have more goodliness as a class. Then how shall we get our education? By honestly seeking it, irrespective of how much it will cost. Although we claim to be Christians, we are almost to the last man of us playing Jew,—trying to buy cheap and sell dear. *E* (Greek,) out of, and *duco* (Latin,) to lead—a hybrid—a Greek-Latin mule that we are riding on. No wonder we tip over the fence, first one way and then another, half milk and half water. Give me hungry students. I can talk to them as far as I can see them. What we need is soul to soul communion, mind to mind interpenetration like the interpenetration of gases, until every one shall be satisfied and say *yes* from the bottom all the way up. When we know that the inquiries that arise in our own minds are satisfied, we are educated, and not till then.

Dr. Osmond. In spite of the poor compensation which professors receive, there are many who glory in the title. Many aspire to it who illy deserve it. Is in favor of a national board for the examination of students and also of professors. A school is established by a few men who get together and obtain a charter; the professors are not appointed by the trustees, but the trustees by the professors; as a general thing, the trustees are simply a farce. Though the professor is not compensated, he stands before the public as a professor, and obtains better fees in consequence. Students are obtained by circulars, and by conferring honorary degrees to secure the influence of the recipients. In some institutions the professors buy in the stock and obtain control, in order that they may be everlasting professors in it.

In many cases the diplomas are bought. Let them be given to those that deserve them, and be without price and above suspicion. The professors should not examine the students; a board of the national Government should examine both them and the professors. Some of the worst quacks are graduates,—laughing-gas and rubber quacks. One graduated, to the speaker's knowledge, after a three-weeks' session, who had never pulled a tooth in his life until ten months before; in four months he sold twenty-one pwts. of gold which he had taken from teeth he had extracted. Over such shops should be written Dante's motto, "All hope abandon, ye who enter here."

Dr. Watt. Is not now connected with any college, though he had once been, both as student and teacher; in that institution great care was taken that the students should give evidence of moral character and manly disposition. Some proved derelict afterwards,—but there is no professional or classical institution that has not had the same experience. One year the questions were printed, and the student had no opportunity of knowing what they were to be, and the examinations were lengthy and rigid. His alma mater has been about as much disgraced as any; one of its graduates is advertising to do rubber work for eight dollars.

Dr. Rehwinkel. Dental colleges have been very severely criticised, but do we do our duty as educators, as a body, and in societies? These last alone have been the means of bringing our profession up to its present standpoint, though many go away from them disappointed. We are all of us teachers in the true sense. A different course will be pursued by dental colleges at some future day. They will be made to feel that they have given grounds for disappointment, and the profession will see that they have failed to encourage and sustain them. Preceptors are remiss, and the colleges, have to go down and commence from the very foundation for want of a preliminary education, and in many cases the student must unlearn what he has learned. In Germany, public opinion is much divided; some insist that the student shall go to a medical college and become an educated physician. There is a sharply drawn distinction between those who have diplomas and those who have not. *Dr.* A. may be no better dentist or operator than *Mr.* B., yet the latter is cut off from speaking in an assembly of this kind. Others there, especially those who have more thorough knowledge of this country, favor the American system, and point with force and truth to the fact that, so far as accomplished operators or manipulators are concerned, the Americans are far in advance, and claim that they stand as high in scientific attainments.

Dr. Butler offered the following, which was adopted:

*Resolved*, That it is the opinion of the American Dental Association, that the time has fully come when degrees should not be conferred by dental colleges upon any student who has attended but one course of lectures.

Adjourned.

*Afternoon Session.*

Met pursuant to adjournment.

On motion of Dr. Judd, it was resolved that the "Transactions" of former years now on hand be given to members of the profession who may call for the same, and the remainder be donated to local societies who will pay the cost of transportaion; (Drs. L. D. Shepard, Boston; W. H. Goddard, Louisville; A. M. Leslie & Co., St. Louis; and M. S. Dean, Chicago, may be applied to for these volumes of "Transactions."

The Nominating Committee reported the following list of standing committees, who were unanimously elected:

*Physiology.*—J. H. McQuillen, E. S. Gaylord, J. R. Walker.

*Pathology*—H. Judd, L. D. Shepard, J. S. Knapp.

*Histology and Microscopy.*—J. Taft, E. D. Swain, W. H. Jackson.

*Chemistry.*—H. A. Smith, S. B Palmer, J. S. Cassidy.

*Therapeutics.*—E. A. Bogue, W. O. Kulp, C. C. Canine.

*Operative Dentistry.*—G. H. Cushing, C. S. Stockton, S. H. McCall.

*Mechanical Dentistry.*—F. H. Rehwinkel, J. F. Canine, J. Johnston.

*Dental Education.*—G. W. Keely, A. H. Brockway, S. Welchens.

*Dental Literature.*—J. S. Knapp, L. G. Noel, C. D. Cook.

*Etiology.*—H. S. Chase, E. C. Hawxhurst, C. S. Smith.

*Prize Essays.*—I. Forbes, G. L. Field, R. B. Donaldson.

The regular order of business was resumed, and the subject of dental education was further discussed.

Dr. Horton. We should not criticise too harshly in this matter except in the spirit of liberality. Daniel Webster, it is said, tore his diploma to pieces upon the platform at his graduation, and told the faculty that he would not build upon it, but would build npon his own foundation. We know what his reputation was. It is not necessary that we should have a diploma; if we have the knowledge we need not fear being recognized. Will stand with Dr. Atkinson with the dental profession. The dentists will stand favorably as compared with medical men everywhere.

Dr. Judd. If we are not specialists of medicine, what are we? As to whether we are recognized as we deserve, has views different from those that have been expressed. When we take a fair view of the subject, we have no right to complain. The National Medical Association requires that its delegates shall be medical men; if that body should see fit to send a delegate here who was not a practicing dentist, we should reject him. A few journals have sought to throw discredit upon the dental profession, but the great body of physicians recognize dentists to the fullest extent they have a right to demand. If they recognized all of us as polished physicians, they would make a mistake; but they are anxious that we should educate ourselves generally, and stand upon the same footing as oculists and other true specialists. If we desire to be specialists, we should make ourselves such; educate ourselves as medical men; and the best way to do it is in a medical college. The medical profession appropriates the best materials as teachers, and if we desire that education we must get it where the best teachers are employed. If there is a prejudice against us, how shall we eradicate it better than by allowing our students to sit side by side with them, and show that we are their equals? The colleges have done a great work, but are not all that they ought to be. This discussion will have a great deal to do in shaping their future course, and will improve them. This Association should keep a close eye upon these institutions, and eventually control them. Some schools are kept up for the benefit of the professors, and therefore the control of the institutions should rest with the profession at large.

Dr. Bogue offered the following:

*Resolved*, That it is the sense of this Association that no dental student should be graduated by any dental college without at least three years instruction, including private pupilage and college instruction; the latter should in no case embrace less than two regular courses.

*Resolved*, That this Association suggest to the different colleges of the country to appoint a common Examining Committee, consisting of five members, none of whom shall be in connection with any dental college, whose duty it shall be to examine all applicants for graduation, and decide upon the same.

Dr. Judd offered the following;

*Resolved*, That this Association recommends to all local societies the adoption of rules prohibiting their members from taking students for a less period than three years, or for such a time as will complete a three years' pupilage.

These, by vote, were laid over one year.

The committee to co-operate with Dr. S. S. White in defending the rights of dentists against the Vulcanite Company, being called upon, Dr. Judd made a brief report. The thanks of the Association were voted to the committee, and it was continued.

Dr. Knapp offered the following:

*Resolved*, That the thanks of this Association are due, and are hereby tendered to the Michigan State Dental Protective Union for the energetic, and so far successful manner in which its officers have resisted the claims of Josiah Bacon and the Goodyear Dental Vulcanite Company. Carried.

Dr. Allport offered the following amendment to the Constitution:

"That the American Dental Association will hereafter admit no delegate who shall enter the profession without first having graduated at some reputable medical or dental college."

Laid over till next year.

The committee to prepare a circular for the information of the public on the subject of "Care of the Teeth," requested further time. Granted.

Dr. McQuillen gave notice of a proposed change in the By-laws, making twenty-four members a quorum.

The newly-elected officers were then inducted into office by Drs. Morgan and Allport.

The report of the Committee on "Mechanical Dentistry," written by Dr. Swain, was then read. The following is a synopsis;

The report noticed no improvement during the year, and indeed stated that the novelties of the year were almost utterly worthless, of which vulcanizable gutta-percha was quite so. The metallic alloys have failed to come into general use. Rose pearl, owing to its complicated method of manipulation, has not grown into general favor, although it appears to possess the properties of a durable base. Celluloid has grown greatly in favor on account of its simplicity of manipulation, and is quite extensively used.

It is a better conductor of heat and cold than rubber, and gives better adaptation to the parts. Aluminium, which promised so well, has been proved to lack durability; in alkaline mouths it is easily destroyed,—blister-like spots appear in contact with the mucous membrane, which soon become holes. It may be owing entirely to impurity of the metal. If this is the case, we have a metallic base in many respects superior to all others. Many of the objections to rubber may be obviated by using the black variety, which contains no vermilion, and is stronger. No improved machinery for the laboratory is noticed except the steam celluloid apparatus, and Hopkin's regulator for the vulcanizer, which automatically turns off the gas at any desired length of time. The report entered a protest against the proposed separation of the mechanical from the operative department of dentistry, particularly the plan of leaving it optional with the student in college whether he shall become proficient in the mechanical department. The profession is desirous of improving in this direction, as is shown by articles in the journals on improved methods of setting pivot teeth, and the efforts to produce a substitute for vulcanite. We are dissatisfied, and there is no reason why the colleges should at this time throw obstacles in the way of advance. The two branches, except in large cities, and even there only partially, can never be separated by the general practitioner. The college which graduates a student incapable in this direction, not only does the individual an injury, but possibly a community. An ambition to excel in this direction would soon make an advance. This department is not to be elevated by being made a specialty. The man who cares for the teeth of a family is the one to replace them when lost. The teachers in our schools, and those who pride themselves on their ability to restore, are the men to elevate this almost dead arm of dentistry.

The hour for adjournment having arrived, the remainder of the subjects were passed, and the association adjourned to meet at Niagara Falls, on the first Tuesday of August, 1875.

